# Publishing in the Era of Artificial Intelligence: What Do Medical Research Journals Expect?

**DOI:** 10.7759/cureus.104955

**Published:** 2026-03-09

**Authors:** Irrum Afzal, Sarkhell Radha

**Affiliations:** 1 Trauma and Orthopaedics, Croydon University Hospital, London, GBR

**Keywords:** artificial intelligence, artificial intelligence in medicine, medical research, publishing, research & development

## Abstract

Artificial intelligence (AI) is now embedded across medical research and practice, reshaping how evidence is generated and evaluated. Rather than lowering standards, AI has intensified editorial expectations within medical journals. Editors prioritise clinically meaningful knowledge over technical novelty, demanding a clear demonstration of how AI augments clinical reasoning and improves patient outcomes. Transparency in AI use, rigorous methodology, bias assessment, and external validation are increasingly essential. As generative AI normalises polished writing, originality is judged by intellectual and clinical contribution rather than style. Ultimately, journals seek responsible, reproducible, and ethically grounded AI-enabled research that advances patient care and public trust.

## Editorial

Artificial intelligence (AI) is no longer a future promise in medicine; it is a present reality. Across radiology, pathology, clinical decision support, genomics, population health and healthcare research, AI-driven tools are reshaping how medical knowledge is generated, analysed and applied [1-3]. As a result, medical research journals now occupy a critical gatekeeping role at the intersection of technological innovation, scientific evidence and patient safety. In this evolving landscape, editorial priorities are not diminishing but becoming increasingly rigorous. Rather than obscuring expectations, the rise of AI has clarified what journals are truly seeking.

Medical research journals are not publishing algorithms; they are publishing knowledge intended to improve health outcomes. While AI systems excel at processing large datasets and identifying complex statistical patterns, journals continue to prioritise research grounded in clinical reasoning and human judgement [1,4]. Editors increasingly expect authors to demonstrate how AI augments rather than replaces clinical expertise, often through study designs that compare clinician performance alone, AI performance alone, and clinician-with-AI decision making. Studies that focus narrowly on technical performance metrics, such as accuracy or area under the curve, without articulating clear clinical relevance are often viewed as insufficient [5]. Instead, journals favour work that explains how AI-driven findings translate into improved diagnosis, prognosis, workflow efficiency or patient safety [6].

One of the most significant shifts in editorial standards is the heightened demand for transparency regarding AI use. Medical journals and the International Committee of Medical Journal Editors (ICMJE) increasingly require authors to disclose whether AI tools were used in data analysis, image interpretation, predictive modelling or manuscript preparation [7,8]. This expectation aligns with broader principles of research integrity and authorship accountability. Undisclosed or poorly described AI use raises concerns about fabricated references, unverifiable analyses, and hidden biases [9]. Transparent reporting enables peer reviewers to assess methodological validity and allows readers to understand the provenance of findings, an essential requirement when research may influence clinical decision-making [10]. This is particularly important in the context of AI-generated content, where unreported AI hallucinations such as fabricated references, incorrect statistical values or non-existent clinical trial data may inadvertently compromise the reliability and reproducibility of medical research [10].

The integration of AI has risen, rather than lowered, expectations for methodological rigour. Journals now scrutinise data origin, source, pre-processing steps and model assumptions with increasing intensity [11]. Particular attention is paid to bias, fairness and generalisability, especially when models are trained on retrospective or single-centre data [12]. Editorial standards increasingly prioritise external validation, prospective evaluation and real-world clinical testing over retrospective performance alone [13]. Reporting frameworks such as TRIPOD-AI (Transparent Reporting of a multivariable prediction model for Individual Prognosis Or Diagnosis for Artificial Intelligence) and CONSORT-AI (Consolidated Standards of Reporting Trials for Artificial Intelligence) reflect this broader shift toward reproducibility and clinical applicability [14,15].

As generative AI makes fluent scientific writing widely accessible, journals are also redefining how originality is assessed. Polished prose is no longer a reliable indicator of scholarly contribution. Instead, originality is judged by the intellectual contribution of the work, novel insights into disease mechanisms, meaningful changes to clinical pathways or improved understanding of health system performance [16]. While AI-assisted writing may enhance clarity, editors report growing concern over manuscripts that are stylistically uniform yet conceptually shallow [9]. Consequently, renewed emphasis is placed on coherence, argumentation and a clearly articulated scholarly voice.

Ethical considerations are integral to the editorial evaluation of AI-based medical research. Beyond traditional concerns such as informed consent, data privacy and algorithmic opacity, journals increasingly assess whether AI applications may amplify bias, exacerbate health inequities or erode trust in healthcare systems [17-19]. Medical journals are explicit that AI tools cannot be credited as authors; responsibility for the accuracy, interpretation and ethical integrity of a manuscript rests entirely with human researchers [7,8]. Authors are expected to verify all AI-assisted outputs, including references, statistical analyses and narrative claims.

Ultimately, medical research journals exist to support evidence-based practice. In the era of AI, this mission remains unchanged but has become more demanding. Journals increasingly prioritise reproducible, clinically actionable research over short-lived technical demonstrations [11,20,21]. Open data practices, shared code and clear documentation are widely viewed as indicators of scientific seriousness. Editors value interdisciplinary collaboration, recognising that responsible AI innovation in medicine requires sustained engagement among clinicians, data scientists, ethicists and health systems researchers [2,6,22]. However, editors also need to be mindful of AI-crafted publications, as these become increasingly common, often characterised by highly fluent but formulaic language, repetitive transitional phrases, generic academic vocabulary, consistent sentence structure and sometimes unusually low similarity scores in tools such as Turnitin.

In conclusion, in this new era, the defining question for journals is not whether AI was used, but whether its use meaningfully advances patient care, medical understanding and public trust. Editors are not seeking faster publications or automated outputs; they are seeking research that combines computational power with clinical insight, methodological rigour, ethical responsibility and accountability. Ultimately, the value of AI in medical publishing will be judged not by its novelty, but by how effectively it advances reliable scientific knowledge, improves health outcomes and safeguards the integrity of the scientific literature.
